# Street Sign Recognition Using Histogram of Oriented Gradients and Artificial Neural Networks

**DOI:** 10.3390/jimaging5040044

**Published:** 2019-04-03

**Authors:** Kh Tohidul Islam, Sudanthi Wijewickrema, Ram Gopal Raj, Stephen O’Leary

**Affiliations:** 1 Department of Surgery (Otolaryngology), Faculty of Medicine, Dentistry and Health Sciences, University of Melbourne, Melbourne, Victoria 3010, Australia; 2 Department of Artificial Intelligence, Faculty of Computer Science and Information Technology, University of Malaya, Kuala Lumpur 50603, Malaysia

**Keywords:** street sign, autonomous vehicle navigation, computer vision, artificial neural networks

## Abstract

Street sign identification is an important problem in applications such as autonomous vehicle navigation and aids for individuals with vision impairments. It can be especially useful in instances where navigation techniques such as global positioning system (GPS) are not available. In this paper, we present a method of detection and interpretation of Malaysian street signs using image processing and machine learning techniques. First, we eliminate the background from an image to segment the region of interest (i.e., the street sign). Then, we extract the text from the segmented image and classify it. Finally, we present the identified text to the user as a voice notification. We also show through experimental results that the system performs well in real-time with a high level of accuracy. To this end, we use a database of Malaysian street sign images captured through an on-board camera.

## 1. Introduction

Automatic vehicle navigation is typically performed using the global positioning system (GPS). However, GPS-based navigation could become unreliable due to several reasons, such as, interference from other systems, issues with signal strength, limitations in receiver sensitivity, and unavailability of maps. Therefore, it is advantageous to have a back-up system to take over the navigation process in case of such issues. Vision-based navigation is an alternative solution, in which street sign recognition plays a major role. Also, due to improper maintenance and the use of non-contrasting colours, people with vision impairments may find it difficult to read street signs. Automatic street sign recognition can be used to aid such individuals as well.

Street signs typically contain alphanumerical characters, the identification of which has received much focus in recent years, specifically in the context of scanned books and documents [1,2,3,4]. Street sign recognition, like other similar problems such as license plate and transport route recognition, typically consists of three stages: (i) extraction of the region of interest (ROI) from the image, (ii) segmentation of characters, and (iii) character recognition [5]. For ROI extraction and character segmentation, researchers use many different methods, the majority of which are based on image thresholding. Classifiers are usually trained for the character recognition task.

A street name detection and recognition method in the urban environment was developed by Parizi et al. [6]. First, they used Adaboost [7] to detect the ROI. Then, they used histogram-based text segmentation and scale-invariant feature transform (SIFT) [8] feature matching for text image classification. An approach for text detection and recognition in road panels was introduced by Gonzalez et al. [9]. For character text localisation, they used the maximally stable extremal regions (MSER) algorithm [10]. An algorithm based on HMM was used for word recognition/classification.

In license plate recognition, some researchers used adaptive boosting in conjunction with Haar-like features for training cascade classifiers [11,12,13]. Others used template matching to recognise number plate text [14,15,16,17]. Dia et al. [18] proposed a method for character recognition in license plates which was based on histogram projection, fuzzy matching, and dynamic programming. In Hegt et al. [19], a feature vector was generated from the Hotelling transform [20] of each character. The charters were then classified based on a distance measure between their Hotelling transformed counterparts and Hotelling transformed prototypes. Others employed feature extraction methods (such as, (LBP) [21], Haar-like [22], and bag-of-features (BoF) [23]) coupled with classifiers (such as artificial neural networks (ANN) [24,25,26], support vector machines (SVM) [27], and hidden Markov models (HMM) [28]) for character recognition. Similarly, Leszczuk et al. [29] introduced a method for the recognition of public transportation route numbers based on optical character recognition (OCR). Intelligence transportation systems use a similar process of segmenting the region of interest from and images and identifying text using optical character recognition to read traffic signs [1,2,3,5,29,30,31,32,33,34,35].

In this paper, we discuss the development of an intelligent identification and interpretation system for Malaysian street signs. The system consists of the following steps: (1) real-time capture of images using a digital camera mounted on the dashboard of a vehicle, (2) segmentation of the street sign from each frame captured by the camera, (3) extraction of characters from the segmented image, (4) identification of the street sign, and (5) presentation of the identified street sign as a verbal message. We showed through experimental results that the proposed method is effective in real-time applications and comparable with other similar existing methods. The remainder of the paper is organised as follows. The proposed methodology for street sign recognition and interpretation is described in Section 2. The experimental setup and results (including performance comparisons) are discussed in Section 3. Section 4 concludes the paper.

## 2. Methodology

**Figure 1:**
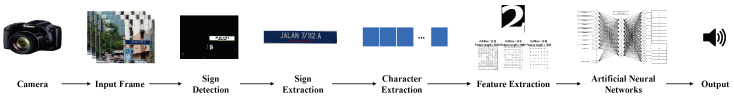
Overview of the proposed system for automatic street sign identification and interpretation.

This section describes the development of the street sign recognition system for Malaysian street signs. An overview of the system is shown in Figure 1 and the functionality of each step is discussed in the following sub-sections.

### 2.1. Acquisition of Images

**Figure 2:**
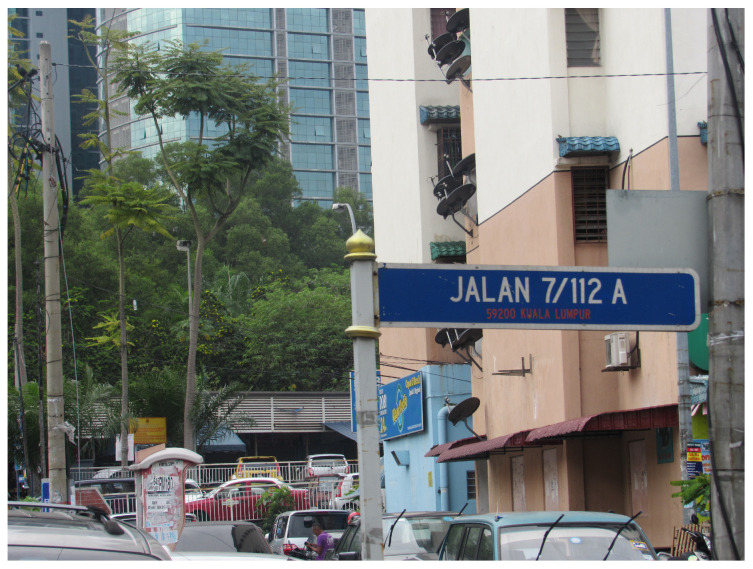
An example of a Malaysian street sign.

Images of Malaysian street signs were captured through a digital camera mounted on the dashboard of a vehicle driving through the streets of Kuala Lumpur. Specifications of the camera used for the image acquisition are shown in Table 1. Over two thousands images of street signs were obtained using this process. Figure 2 shows an example of a Malaysian street sign thus captured.

### 2.2. Extraction of the Region of Interest

**Figure 3:**
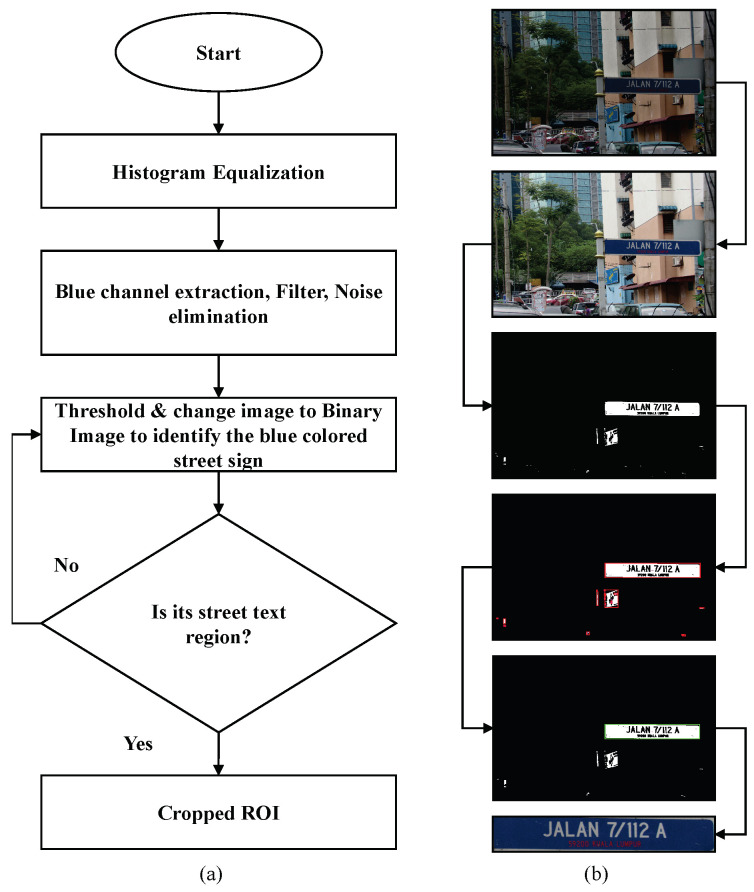
Extraction of the region of interest: (**a**) block diagram of the algorithm and (**b**) results at each step.

We pre-processed the obtained images to extract the region of interest (i.e., the area that contains the street sign). First, a histogram equalisation was performed on the image frame to improve contrast. Next, the blue channel of the image was extracted and noise was removed from it (salt and pepper noise removal and median filtering). Then, using a simple theresholding method, it was binarised to identify the blue coloured street sign. The resulting image contained all possible objects for the street sign. The objects were identified using a blob detection algorithm. An object measurement algorithm was used to find the most likely candidate for the street sign using the object’s height to width ratio. This was then used as a mask to extract the region of interest from the histogram equalised image (output of the first step of this algorithm). Figure 3 illustrates this process, with Figure 3a showing the block diagram of the process and Figure 3b showing the results at each step for an example image.

### 2.3. Extraction of Text

**Figure 4:**
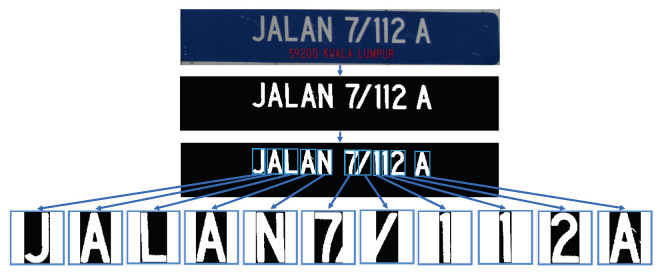
Extraction of text from the street sign.

From the region of interest obtained in the previous step, we extracted the text of the street sign. First, the street sign was converted to greyscale. Then, it was binarised using thresholding. A 3×3 median filter was used for smoothing the binary image and removing small objects. The objects that remained in the binary image were considered to represent a character. Next, the region of interest for each object was extracted and normalised into 56 × 56 pixels. Figure 4 illustrates the text extraction process.

### 2.4. Calculation of Text Features

**Figure 5:**
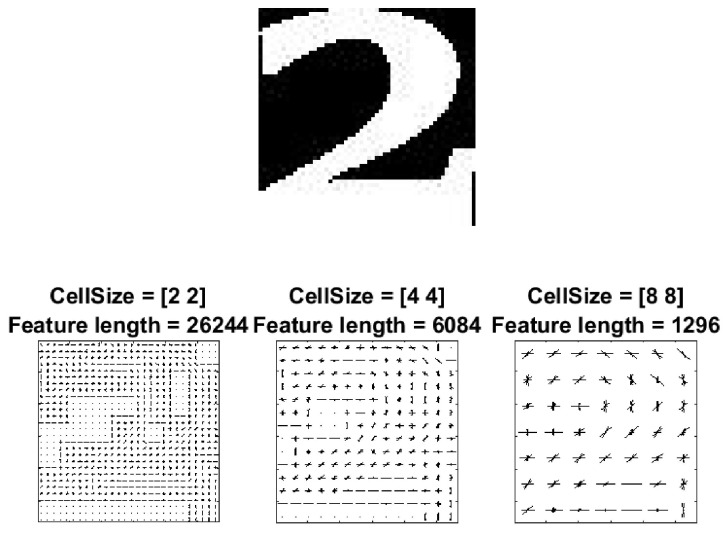
Visualisation of dimension in the histogram of oriented gradients (HOG) feature vector.

The extracted characters then went through a feature calculation process, so that each character could be represented as a set of feature values. Here, we used histogram of oriented gradients (HOG) [36] for this purpose. First, the image was subdivided into smaller neighborhood regions (or ‘cells’) [37]. Then, for each cell, at each pixel, the kernels [−1,0,+1] and ${\left[-1,0,+1\right]}^{T}$ were applied to get the horizontal ($G_{x}$) and vertical ($G_{y}$) edge values respectively. The magnitude and orientation of the gradient were calculated as $M\left(x,y\right)=\sqrt{\left(G_{x}^{2}+G_{y}^{2}\right)}$ and $\theta \left(x,y\right)=tan^{-1}\left(\frac{G_{y}}{G_{x}}\right)$, respectively. Histograms of the unsigned angle ($0^{{}^{\circ}}$ to $180^{{}^{\circ}}$) weighted on the magnitude of each cell were then generated. Cells were combined into blocks and block normalisation was performed on the concatenated histograms to account for variations in illumination. The cell size affected the length of the feature vector, as shown in Figure 5.

In our application, we used a cell size of 4×4 as it provided a good balance between encoded spatial information and feature dimensions, which helps speed up training. Since our images were 56×56 pixels, this gave us 14×14 cells. Block sizes of 2×2 cells were used for the block normalisation with a 50% overlap. This resulted in 13×13×4 normalised histograms. Each histogram had nine bins, and as such, the resulting feature vector for each image had 13×13×4×9=6084 elements.

### 2.5. Character Recognition

**Figure 6:**
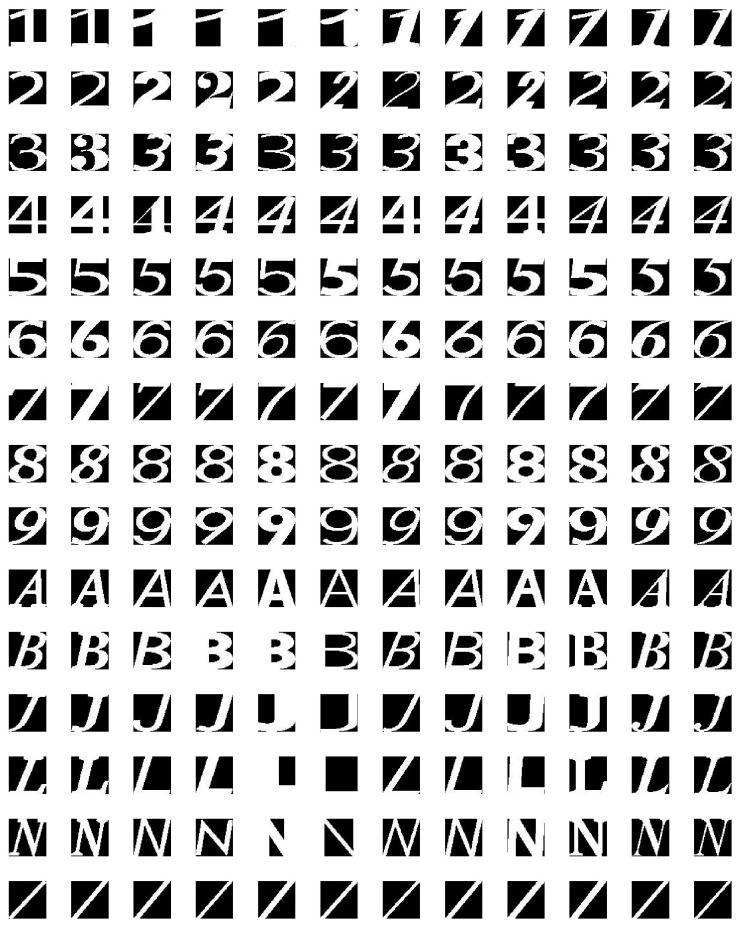
Example training data set.

**Figure 7:**
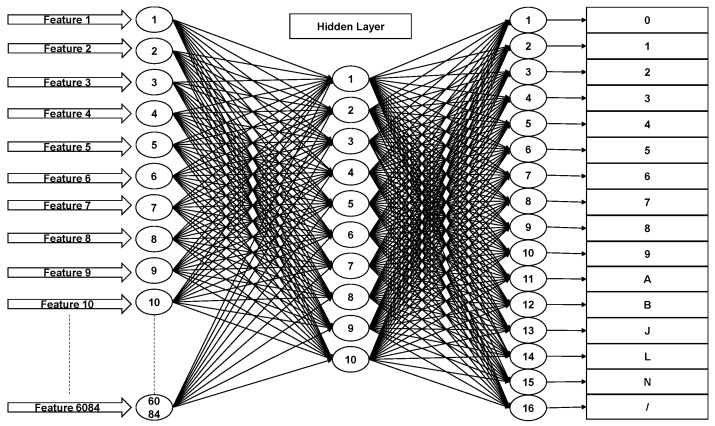
Artificial neural network architecture.

In Malaysia, street signs contain a specific set of 16 characters (‘0’, ‘1’, ‘2’, ‘3’, ‘4’, ‘5’, ‘6’, ‘7’, ‘8’, ‘9’, ‘A’, ‘B’, ‘J’, ‘L’, ‘N’ and ‘/’) as shown in Figure 6. Therefore, we trained a neural network to classify each character into one of 16 classes. It contained one hidden layer with 10 neurons. The input to this classifier was the 6084-element feature vector calculated in the previous step. Back propagation was used to train the neural network. Figure 7 shows the architecture of the neural network classifier. The training dataset contained 200 text images per class, resulting in a total of 3200 training samples. 70% of these samples were randomly allocated to the training set (2240 samples). We used 15% (480 samples) each for validation and testing.

### 2.6. Text to Voice Interpretation

**Figure 8:**
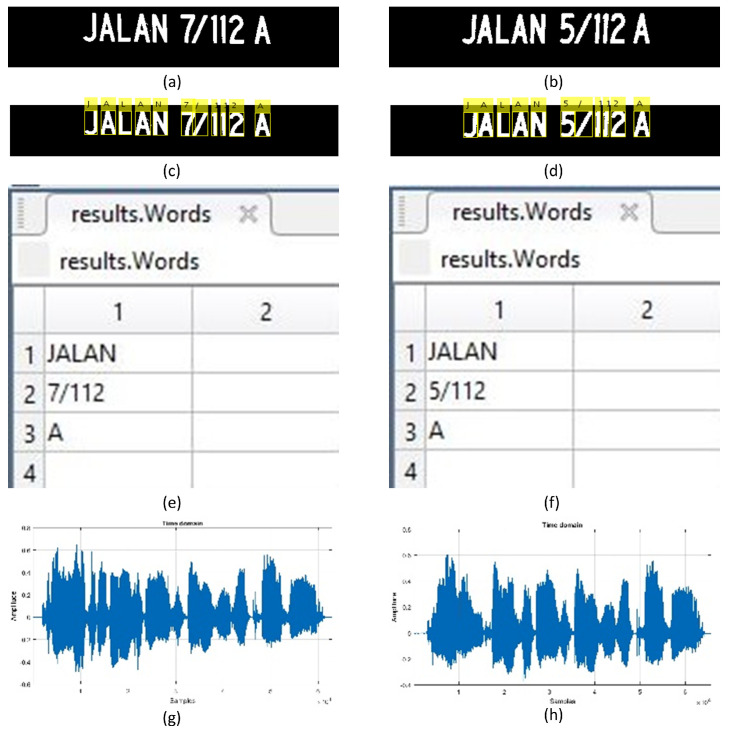
Presentation of recognised street signs: (**a**,**b**) are street signs, (**c**,**d**) are annotated street signs with recognised text, (**e**,**f**) are extracted text words, and (**g**,**h**) are the voice plots.

The proposed system can notify the user of the recognised street signs in two ways. It can either be visualised as text or converted to voice. Figure 8 shows these two forms of output of the street sign recognition system.

## 3. Experimental Results

To conduct the experiments, a Dell latitude E6420 (Dell Inc., Round Rock, TX, USA) computer running windows 10 professional (64-bit) powered by Intel^®^ Core^™^ (Intel, Santa Clara, CA, USA) i5 2.5 GHz processor, and 8 GB of RAM (Dell Inc., Round Rock, TX, USA) was used. The system and related experiments were implemented using MATLAB (R2016a) (MathWorks, Natick, MA, USA) image processing, computer vision, and neural network toolboxes.

### 3.1. Training Performance of the Neural Network

**Figure 9:**
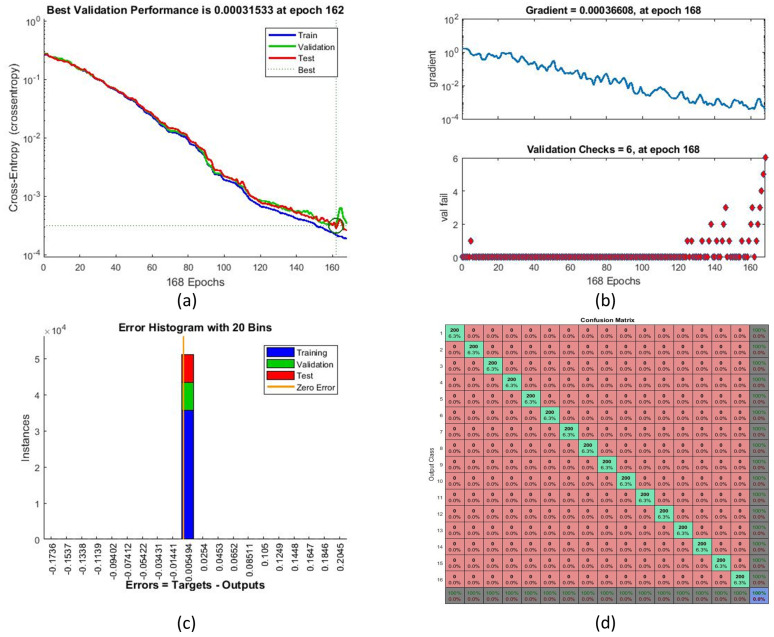
Neural network training performance parameters: (**a**) cross-entropy, (**b**) training state, (**c**) error histogram, and (**d**) overall confusion matrix.

**Figure 10:**
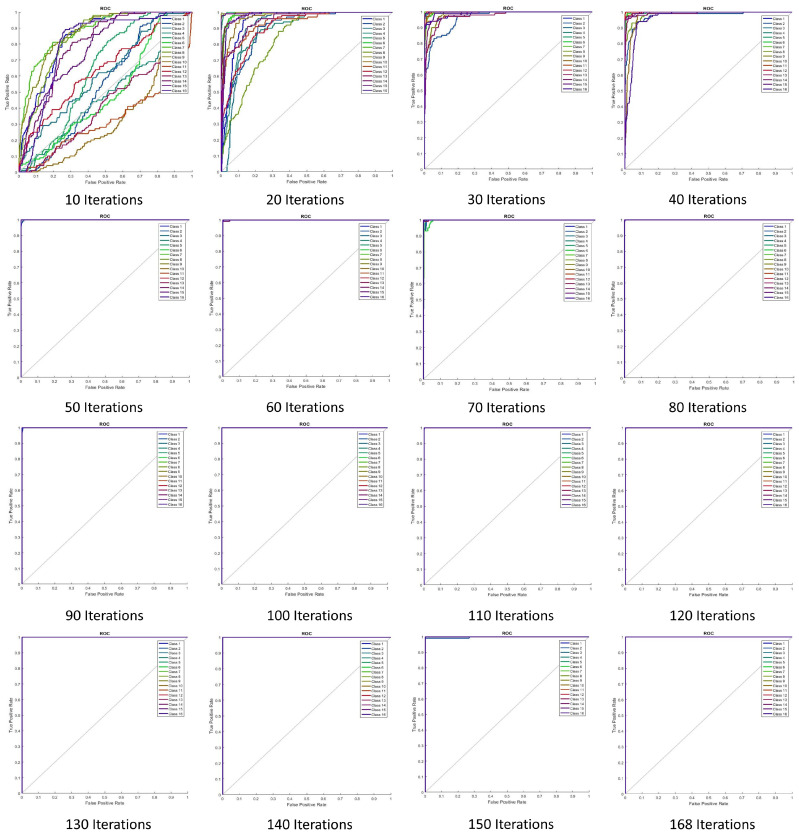
Neural network training performance with respect to the receiver operating characteristic (ROC) curve.

The training performance of the neural network for several training iterations is shown in Table 2. Training number 16 with 168 iterations provided the best error rate (0%), and as such was considered the optimal training number for the proposed system. Figure 9 illustrates the training performance with respect to parameters such as cross-entropy error. Receiver operating characteristic (ROC) curves and the performance of ANN training are shown in Figure 10. Perfect classification results were seen at 168 iterations.

### 3.2. Performance on Testing Data

**Figure 11:**
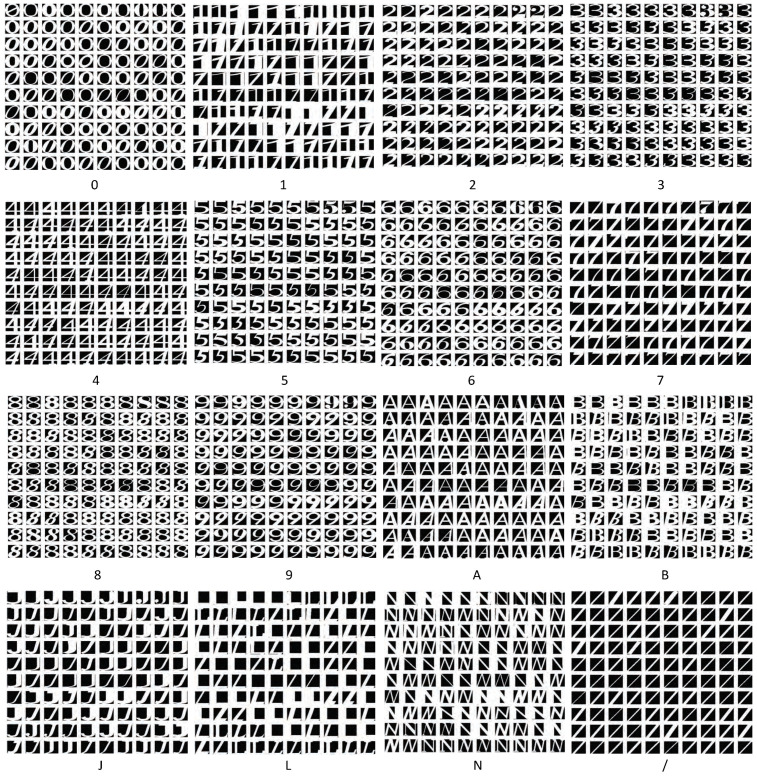
Testing image samples.

Data that had not been used in the training process was used to test the performance of the neural network classifier. The testing dataset was extracted from images of the street sign from Malaysian street signs using the process discussed in Section 2. Each class contained 100 test samples, resulting in a total of 1600 samples (see Figure 11).

**Figure 12:**
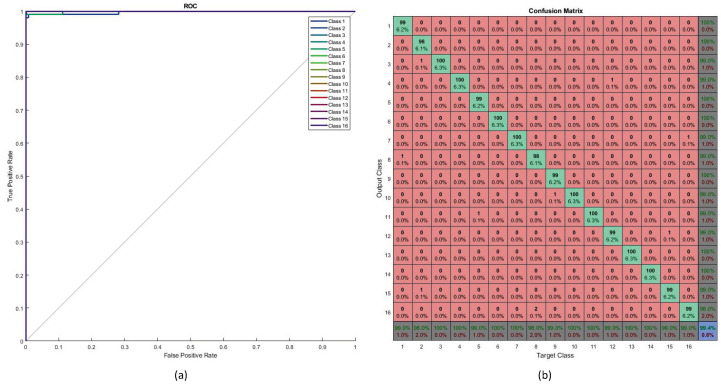
Testing performance, (**a**) testing ROC curve and (**b**) testing confusion matrix.

**Figure 13:**
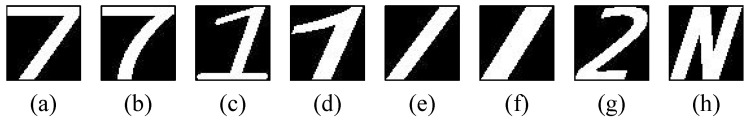
Some images taken from the testing data set that led to misclassifications: (**a**,**b**) are class ‘7’, (**c**,**b**) are class ‘1’, (**e**,**f**) are class ‘/’, (**g**) is class ‘2’, and (**h**) is class ‘N’.

Testing performance is shown in Figure 12. The ROC curve (as shown in Figure 12a) determines the values of the area under curve (AUC) for all testing samples (16 classes). Most of the classes achieved perfect AUC as seen from the figure. The confusion matrix for the testing data is shown in Figure 12b. The overall percentage of correct classification was 99.4%. The highest levels of misclassification were observed in the class pairs of ‘7’ and ‘/’, ‘1’ and ‘7’ and ’2’ and ’N’. Figure 13 shows some characters that led to miscalssifications.

Testing performance with respect to some common metrics, along with how they were calculated based on the the number of true negative (TN), true positive (TP), false negative (FN), and false positive (FP) classifications, is shown in Table 3.

### 3.3. Comparison of ROI Extraction using Different Colour Spaces

**Figure 14:**
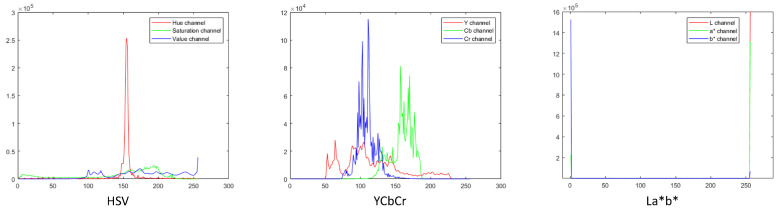
HSV, YCbCr, and CIEL*a*b* colour profiles.

To observe if choice of colour space used in the ROI extraction played a significant role in the performance of our method, we compared the original method based on the RGB colour space with those using other colour spaces such as HSV, YCbCr, and CIEL*a*b* [38]. First, we manually cropped 20 images to extract the ROI. Then, we created the colour profiles for these extracted regions in each colour space. Figure 14 shows the colour profile histograms for HSV, YCbCr, and CIEL*a*b* colour spaces. Next, for each channel in a colour space, we calculated the value range using mean (μ) and standard deviation (σ) as [μ−2σμ+2σ]. This value range was then used to threshold the ROI from the image. Comparison results with respect to the different ROI extraction methods are shown in Table 4. As seen from the comparison results, the choice of colour space does not significantly affect the performance of the system.

### 3.4. Comparison of Different Feature Extraction Methods

To explore the effect of the feature extraction method, we compared the classification performance when using the original HOG features and some others that are often used in the literature: local binary patterns (LBP) [21], Haar-like [22], and bag-of-features (BoF) [23]. We used our ANN as the classifier. We considered accuracy and average time to extract features across all images to be measures of performance in this comparison. Table 5 shows the results. From this comparison, we observe that HOG provides the best accuracy and a comparable level of efficiency.

### 3.5. Comparison with Similar Existing Methods

To evaluate the performance of the proposed method on the Malaysian street sign database, we compared it to some similar existing methods. For example, Kamble et al. [39] discussed handwritten character recognition using rectangular HOG (R-HOG) feature extraction and used a feed-forward neural network (FFANN) and a support vector machine (SVM) for classification. Su et al. [40] also investigated the character recognition task in natural scenes. They used convolutional co-occurrence HOG (CHOG) as their feature extractor and SVM as their classifier. Tian et al. [41] performed text recognition with CHOG and SVM. Boukharouba et al. [42] classified handwritten digits using a chain code histogram (CCH) [43] for feature extraction and a SVM for classification. Niu et al. [44] introduced a hybrid method for recognition of handwritten digits. They used a convolution neural network (CNN) to extract the image features and fed them to a hybrid classifier for classification. Their hybrid classifier contained a CNN and SVM. For the purpose of comparison, we re-implemented these methods and trained and tested them on our database. The pre-processing procedure discussed above was performed to extract text from the images for all compared methods. Table 6 shows the comparison results.

### 3.6. Comparison of Methods with Respect to the MNIST Database

To investigate the transferability of the proposed method, we compared its performance with the above methods on a publicly available text image database. For this purpose, we used the modified national institute of standards and technology (MNIST) database [45]. As this database only contains text images, no pre-processing (as discussed in Section 2.2) was required. As such, only feature extraction and classification methods were considered here. Table 7 shows the comparison results. The execution time here refers to the average time (in seconds) for feature extraction and recognition of a single character. We observed that the method discussed in Niu et al. [44] performed best with respect to classification accuracy. The proposed method was slightly less accurate, but shows the best execution time.

## 4. Conclusions

In this paper, we presented a system for Malaysian street sign identification and interpretation in real-time. The proposed system consisted of a few steps: image acquisition, extraction of the region of interest (i.e., the street sign) from the image, extraction of text, calculation of features (histogram of oriented gradients) from the text, recognition or classification of the text (using a neural network), and the presentation of the identified text visually and verbally. Experimental results showed high performance levels (including when compared to other similar existing methods), indicating that the proposed system is effective in recognising and interpreting Malaysian street signs.

As such, it can be used as an alternative/backup to GPS-based navigation and as an aid for visually impaired individuals. In future work, we will investigate the use of deep learning techniques in the recognition system. We will also explore how this system can be used for identifying street signs in other countries and under difficult imaging conditions (such as low-lit environments at night). We will also extend it to be used in other similar applications such as license plate detection and traffic sign detection. Furthermore, we will also consider methods to possibly improve detection levels, for example, the removal of deformations (such as that caused by perspective projection) as a pre-processing step. 

## Figures and Tables

**Table 1:** Camera specification and image acquisition parameters.

Name	Description
Image acquisition device name	Canon Power Shot SX530 HS
Weather	Daylight, rainy, sunny, cloudy
Capturing period	8 a.m. to 6 p.m.
Background	Complex; not fixed
Horizontal field-of-view	Approximately 75${}^{{}^{\circ}}$
Image dimension	4608 × 3456
Maximum capturing distance	60 m
Street sign condition	Standard, non-standard
Total number of characters	16 (0, 1, 2, 3, 4, 5, 6, 7, 8, 9, A, B, J, L, N, /)
Number of characters acquired per class	300
Total number of character acquired	4800
Total number of training samples	3200
Total number of testing samples	1600

**Table 2:** Neural network training performance.

No. of Training	Iterations	Training Time	Performance	Gradient	Validation Checks	Error (%)
1	10	0:00:01	0.178	0.0472	0	90.50000 × 10${}^{-0}$
2	20	0:00:02	0.0104	0.0433	0	52.25000 × 10${}^{-0}$
3	30	0:01:03	0.0695	0.182	0	15.93749 × 10${}^{-0}$
4	40	0:00:04	0.0625	0.0687	0	15.87500 × 10${}^{-0}$
5	50	0:00:05	0.0168	0.0570	0	3.75000 × 10${}^{-1}$
6	60	0:00:06	0.0170	0.0545	0	4.37500 × 10${}^{-1}$
7	70	0:00:07	0.0158	0.0350	0	2.68750 × 10${}^{-0}$
8	80	0:00:09	0.00842	0.0371	0	4.37500 × 10${}^{-1}$
9	90	0:00:09	0.0067	0.0258	0	1.87500 × 10${}^{-1}$
10	100	0:00:10	0.00162	0.00144	0	6.25000 × 10${}^{-2}$
11	110	0:00:11	0.00256	0.00515	0	6.25000 × 10${}^{-2}$
12	120	0:00:13	0.000735	0.000878	6	6.25000 × 10${}^{-3}$
13	130	0:00:14	0.00423	0.00793	0	2.50000 × 10${}^{-1}$
14	140	0:00:14	0.000813	0.00161	0	6.25000 × 10${}^{-2}$
15	150	0:00:15	0.000869	0.00245	0	1.25000 × 10${}^{-1}$
16	168	0:00:17	4.73 × 10${}^{-7}$	8.68 × 10${}^{-7}$	6	0

**Table 3:** Training performance with respect to common performance metrics.

Evaluation Parameters	Mathematical Equations	Result
Accuracy	$\frac{TP+TN}{TP+FN+FP+TN}$	0.99375
Sensitivity	$\frac{TP}{TP+FN}$	0.99000
Specificity	$\frac{TN}{TN+FP}$	0.99400
Precision	$\frac{TP}{TP+FP}$	0.91667
F-Measure	$2\times \left(\frac{\frac{TP}{TP+FP}\times \frac{TP}{TP+FN}}{\frac{TP}{TP+FP}+\frac{TP}{TP+FN}}\right)$	0.95192
G-Mean	$\sqrt{\frac{TP}{TP+FN}\times \frac{TN}{TN+FP}}$	0.99200

**Table 4:** Comparison results for receiver operating characteristic (ROI) extraction with different colour spaces.

Method	Detection Time (s)	Accuracy (%)
HSV	0.15±0.01	96.980
YCbCr	0.14±0.02	96.698
CIEL*a*b*	0.12±0.03	96.580
Proposed (RGB)	0.13±0.01	96.821

**Table 5:** Performance comparison with different feature extraction methods.

Method	Average Feature Extraction Time (s)	Accuracy (%)
LBP	0.08	97.352
Haar-like	0.07	95.193
BoF	0.12	99.285
Proposed (HOG)	0.10	99.375

**Table 6:** Performance comparison with respect to similar existing methods.

Reference	Features Extraction Method	Classifier (s)	Execution Time (s)	Accuracy (%)
Kamble et al. [39]	R-HOG	FFANN	0.14±0.02	98.718
Kamble et al. [39]	R-HOG	SVM	0.17±0.02	96.523
Su et al. [40]	HOG	SVM	0.19±0.03	94.890
Tian et al. [41]	HOG	SVM	0.18±0.01	94.890
Boukharouba et al. [42]	CCH	SVM	0.18±0.02	98.250
Niu et al. [44]	CNN	CNN + SVM	0.21±0.03	97.325
Proposed	HOG	ANN	0.16±0.01	99.375

**Table 7:** Performance comparison on the modified national institute of standards and technology (MNIST) database.

Reference	Features Extraction Method	Classifier (s)	Execution Time (s)	Accuracy (%)
Kamble et al. [39]	R-HOG	FFANN	0.05	99.134
Kamble et al. [39]	R-HOG	SVM	0.07	98.953
Su et al. [40]	HOG	SVM	0.06	98.400
Tian et al. [41]	HOG	SVM	0.06	98.400
Boukharouba et al. [42]	CCH	SVM	0.06	99.050
Niu et al. [44]	CNN	CNN + SVM	0.07	99.614
Proposed	HOG	ANN	0.05	99.482

## Abbreviations

The following abbreviations are used in this manuscript:

ANN	Artificial neural network
AR	Accuracy rate
AUC	Area under curve
BoF	Bag-of-features
CCH	Chain code histogram
CHOG	Co-occurrence HOG
CNN	Convolution neural network
FFANN	Feed-forward ANN
FN	False negative
FP	False positive
GPS	Global positioning system
HOG	Histogram of oriented gradients
HSV	Hue, saturation, value
LBP	Local binary patterns
MNIST	Modified national institute of standards and technology
MSER	Maximally stable extremal regions
OCR	Optical character recognition
ROC	Receiver operating characteristic
R-HOG	Rectangular HOG
SIFT	Scale-invariant feature transform
SVM	Support vector machine
TN	True negative
TP	True positive

## References

[1] Nagy G. (1992). At the frontiers of OCR. Proc. IEEE.

[2] Casey R.G., Lecolinet E. (1996). A survey of methods and strategies in character segmentation. IEEE Trans. Pattern Anal. Mach. Intell..

[3] Mori S., Suen C.Y., Yamamoto K. (1992). Historical review of OCR research and development. Proc. IEEE.

[4] Plamondon R., Srihari S.N. (2000). Online and off-line handwriting recognition: A comprehensive survey. IEEE Trans. Pattern Anal. Mach. Intell..

[5] Anagnostopoulos C.E., Anagnostopoulos I.E., Psoroulas I.D., Loumos V., Kayafas E. (2008). License Plate Recognition From Still Images and Video Sequences: A Survey. IEEE Trans. Intell. Transp. Syst..

[6] Parizi S.N., Targhi A.T., Aghazadeh O., Eklundh J. (2009). Reading street signs using a generic structured object detection and signature recognition approach. Proceedings of the Fourth International Conference on Computer Vision Theory and Applications.

[7] Freund Y., Schapire R., Abe N. (1999). A short introduction to boosting. J.-Jpn. Soc. Artif. Intell..

[8] Lowe D. Object recognition from local scale-invariant features. Proceedings of the Seventh IEEE International Conference on Computer Vision.

[9] Gonzalez A., Bergasa L.M., Yebes J.J., Almazan J. Text recognition on traffic panels from street-level imagery. Proceedings of the 2012 IEEE Intelligent Vehicles Symposium.

[10] Matas J., Chum O., Urban M., Pajdla T. (2004). Robust wide-baseline stereo from maximally stable extremal regions. Image Vis. Comput..

[11] Kahraman F., Kurt B., Gökmen M. (2003). License Plate Character Segmentation Based on the Gabor Transform and Vector Quantization. Computer and Information Sciences-ISCIS.

[12] Zhang X., Shen P., Xiao Y., Li B., Hu Y., Qi D., Xiao X., Zhang L. License plate-location using AdaBoost Algorithm. Proceedings of the 2010 IEEE International Conference on Information and Automation.

[13] Wu Q., Zhang H., Jia W., He X., Yang J., Hintz T. Car Plate Detection Using Cascaded Tree-Style Learner Based on Hybrid Object Features. Proceedings of the 2006 IEEE International Conference on Video and Signal Based Surveillance.

[14] Sarfraz M., Ahmed M., Ghazi S. Saudi Arabian license plate recognition system. Proceedings of the 2003 International Conference on Geometric Modeling and Graphics.

[15] Gupta P., Purohit G.N., Rathore M. (2014). Number Plate Extraction using Template Matching Technique. Int. J. Comput. Appl..

[16] Mayan J.A., Deep K.A., Kumar M., Alvin L., Reddy S.P. Number plate recognition using template comparison for various fonts in MATLAB. Proceedings of the 2016 IEEE International Conference on Computational Intelligence and Computing Research (ICCIC).

[17] Sharma G. (2018). Performance Analysis of Vehicle Number Plate Recognition System Using Template Matching Techniques. J. Inf. Technol. Softw. Eng..

[18] Dia Y., Zheng N., Zhang X., Xuan G. Automatic recognition of province name on the license plate of moving vehicle. Proceedings of the 9th International Conference on Pattern Recognition.

[19] Hegt H., de la Haye R., Khan N. A high performance license plate recognition system. Proceedings of the 1998 IEEE International Conference on Systems, Man, and Cybernetics.

[20] Sanchez-Marin F.J. (2001). Automatic recognition of biological shapes using the Hotelling transform. Comput. Biol. Med..

[21] Ojala T., Pietikainen M., Maenpaa T. (2002). Multiresolution gray-scale and rotation invariant texture classification with local binary patterns. IEEE Trans. Pattern Anal. Mach. Intell..

[22] Viola P., Jones M. Rapid object detection using a boosted cascade of simple features. Proceedings of the 2001 IEEE Computer Society Conference on Computer Vision and Pattern Recognition.

[23] Harris Z.S. (1954). Distributional Structure. WORD.

[24] Barnouti N.H., Naser M.A.S., Al-Dabbagh S.S.M. Automatic Iraqi license plate recognition system using back propagation neural network (BPNN). Proceedings of the 2017 Annual Conference on New Trends in Information & Communications Technology Applications (NTICT).

[25] Islam K.T., Raj R.G. (2017). Real-Time (Vision-Based) Road Sign Recognition Using an Artificial Neural Network. Sensors.

[26] Islam K.T., Raj R.G., Mujtaba G. (2017). Recognition of Traffic Sign Based on Bag-of-Words and Artificial Neural Network. Symmetry.

[27] Khan M.A., Sharif M., Javed M.Y., Akram T., Yasmin M., Saba T. (2018). License number plate recognition system using entropy-based features selection approach with SVM. IET Image Process..

[28] Llorens D., Marzal A., Palazón V., Vilar J.M. (2005). Car License Plates Extraction and Recognition Based on Connected Components Analysis and HMM Decoding. Pattern Recognition and Image Analysis.

[29] Leszczuk M., Skoczylas L., Dziech A. Simple solution for public transport route number recognition based on visual information. Proceedings of the 2013 Signal Processing: Algorithms, Architectures, Arrangements, and Applications (SPA).

[30] Anthimopoulos M., Gatos B., Pratikakis I. (2013). Detection of artificial and scene text in images and video frames. Pattern Anal. Appl..

[31] Basavanna M., Shivakumara P., Srivatsa S.K., Kumar G.H. (2016). Adaptive Histogram Analysis for Scene Text Binarization and Recognition. Malays. J. Comput. Sci..

[32] Gomez L., Karatzas D. (2016). A fast hierarchical method for multi-script and arbitrary oriented scene text extraction. Int. J. Doc. Anal. Recognit. IJDAR.

[33] Jain A., Peng X., Zhuang X., Natarajan P., Cao H. Text detection and recognition in natural scenes and consumer videos. Proceedings of the 2014 IEEE International Conference on Acoustics, Speech and Signal Processing (ICASSP).

[34] Mammeri A., Khiari E., Boukerche A. Road-Sign Text Recognition Architecture for Intelligent Transportation Systems. Proceedings of the 2014 IEEE 80th Vehicular Technology Conference (VTC2014-Fall).

[35] Wang K., Babenko B., Belongie S. End-to-end scene text recognition. Proceedings of the 2011 International Conference on Computer Vision.

[36] Dalal N., Triggs B. Histograms of oriented gradients for human detection. Proceedings of the 2005 IEEE Computer Society Conference on Computer Vision and Pattern Recognition.

[37] Takahashi K., Takahashi S., Cui Y., Hashimoto M. (2014). Remarks on Computational Facial Expression Recognition from HOG Features Using Quaternion Multi-layer Neural Network. Engineering Applications of Neural Networks.

[38] Smith A.R. (1978). Color gamut transform pairs. ACM SIGGRAPH Comput. Graph..

[39] Kamble P.M., Hegadi R.S. (2015). Handwritten Marathi Character Recognition Using R-HOG Feature. Procedia Comput. Sci..

[40] Su B., Lu S., Tian S., Lim J.H., Tan C.L. Character Recognition in Natural Scenes Using Convolutional Co-occurrence HOG. Proceedings of the 2014 22nd International Conference on Pattern Recognition.

[41] Tian S., Lu S., Su B., Tan C.L. Scene Text Recognition Using Co-occurrence of Histogram of Oriented Gradients. Proceedings of the 2013 12th International Conference on Document Analysis and Recognition.

[42] Boukharouba A., Bennia A. (2017). Novel feature extraction technique for the recognition of handwritten digits. Appl. Comput. Inform..

[43] Iivarinen J., Visa A.J.E., Casasent D.P. (1996). Shape recognition of irregular objects. Intelligent Robots and Computer Vision XV: Algorithms, Techniques, Active Vision, and Materials Handling.

[44] Niu X.X., Suen C.Y. (2012). A novel hybrid CNN–SVM classifier for recognizing handwritten digits. Pattern Recognit..

[45] Lecun Y., Bottou L., Bengio Y., Haffner P. (1998). Gradient-based learning applied to document recognition. Proc. IEEE.

